# Attenuated initial serum ferritin concentration in critically ill coronavirus disease 2019 geriatric patients with comorbid psychiatric conditions

**DOI:** 10.3389/fpsyt.2022.1035986

**Published:** 2022-11-09

**Authors:** Osama A. Abulseoud, Asmaa Yehia, Claudine J. Egol, Victor N. Nettey, Mohamed Aly, Yihuai Qu, Aaron B. Skolnik, Marie F. Grill, Ayan Sen, Terry D. Schneekloth

**Affiliations:** ^1^Department of Psychiatry and Psychology, Mayo Clinic Arizona, Phoenix, AZ, United States; ^2^Department of Neuroscience, Mayo Clinic Graduate School of Biomedical Sciences, Collaborative Research Building (CRB), Scottsdale, AZ, United States; ^3^Department of Medical Physiology, Faculty of Medicine, Mansoura University, Mansoura, Egypt; ^4^Department of Cardiothoracic Surgery, Mayo Clinic Arizona, Phoenix, AZ, United States; ^5^Alix School of Medicine at Mayo Clinic, Phoenix, AZ, United States; ^6^Department of Critical Care, Mayo Clinic Arizona, Phoenix, AZ, United States; ^7^Department of Neurology, Mayo Clinic Arizona, Phoenix, AZ, United States

**Keywords:** ferritin, COVID-19, delirium, critical illness *mortality, geriatric, psychiatric disorders

## Abstract

We examined the effects of psychiatric comorbidity, sex, and ICU admission on serum ferritin concentration in 628 elderly patients (79.7 ± 8.5 years) with positive SARS-CoV-2 PCR test. Hospitalization was required in 96% of patients and 17% required ICU admission. Patients with COVID-19 and psychiatric comorbidities (*n* = 212) compared to patients without psychiatric comorbidities (*n* = 416) had significantly lower ferritin concentration (570.4 ± 900.1 vs. 744.1 ± 965, *P* = 0.029), a greater incidence of delirium (22.6 vs. 14.4%, *P* = 0.013) and higher mortality (35.3 vs. 27.6%, *P* = 0.015). Furthermore, we found significant effects for sex (*P* = 0.002) and ICU admission (*P* = 0.007). Among patients without comorbid psychiatric conditions, males had significantly higher ferritin compared to females (1,098.3 ± 78.4 vs. 651.5 ± 94.4, *P* < 0.001). ICU patients without comorbid psychiatric conditions had significantly higher serum ferritin compared to ICU patients with comorbid psychiatric conditions: (1,126.6 ± 110.7 vs. 668.6 ± 156.5, *P* < 0.001). Our results suggest that the presence of comorbid psychiatric conditions in elderly patients with COVID-19 is associated with higher rates of delirium and mortality and lower ferritin levels during severe illness. Whether high serum ferritin is protective during severe infection requires further investigation.

## Introduction

Illness severity and mortality rates are high among geriatric patients with coronavirus disease 2019 (COVID-19) caused by severe acute respiratory syndrome coronavirus 2 (SARS-CoV-2) (1–3). Several factors contribute to this age-related vulnerability such as frailty and medical comorbidity (4–6). One of the medical defense mechanisms that may be impaired in elderly patients is the ability to augment the synthesis of ferritin under conditions of increased cellular oxidative stress, as seen during SARS-CoV-2 infection (7).

Ferritin is a protein synthesized by hepatocytes, macrophages, oligodendrocytes, and other cells to store excess cellular iron and to sequester iron rapidly in response to oxidative stress, to prevent the formation of reactive oxygen species (8–10). During infection, increased ferritin production represents an important host defense mechanism that deprives pathogens of iron necessary for replication and limits the production of free radicals (11).

Serum ferritin is a non-specific acute phase protein (12) that is elevated in many different conditions including inflammation (11, 13–17), malignancy (16, 18, 19), and certain autoimmune diseases (20–22). In COVID-19 patients, higher serum ferritin levels are reported in hospitalized (23, 24), critically ill patients (25–27) with more severe illness (23, 28–31), longer length of hospital stay (32) and in non-survivors (1, 25, 33–39). In addition, one study screened adult COVID-19 survivors (*n* = 109) for depression and anxiety using Beck Depression and Beck Anxiety Inventories (BDI, BAI) (40, 41) on the 15th post-hospitalization day, and reported significant positive correlation between BAI scores and ferritin levels in patients with anxiety (42).

In fact, patients with psychiatric conditions, regardless of COVID-19 infection status, have abnormalities in serum ferritin. High serum ferritin levels were reported in patients with major depression (*n* = 15) compared to patients without major depression (*n* = 92) (43), in patients with post-stroke depression compared to stroke patients without depression (44), in depressed patients with melancholia compared to those with simple major depression and normal controls (45), and in first episode (*n* = 23) and antidepressant-naïve depressed patients (*n* = 15) compared to healthy controls (*n* = 63) (46).

Other studies have reported opposite findings: decreased serum ferritin levels associated with depression. In a large-scale study from Germany, patients with major depression (*n* = 619) had lower ferritin levels compared to patients without major depression (*n* = 3,562) (log-transformed mean, 4.08 vs. 4.2; *P* = 0.003). Among males with cardiovascular disorders (congestive heart failure or hypertension), those with comorbid depression had lower levels of ferritin compared to patients without comorbid depression (47). In another study, depressed patients (*n* = 67) had lower serum ferritin compared to healthy controls (*n* = 125) (48). Moreover, low ferritin was reported in women who developed postpartum depression (*n* = 65) with a strong association between ferritin concentration and postpartum depressive symptom severity (49). Furthermore, ferritin levels were significantly lower in schizophrenic patients with (*n* = 30) and without (*n* = 30) akathisia compared to the control group (*n* = 30) (50) and also in bipolar manic patients compared to bipolar euthymic patients and to healthy controls. Still other studies found no significant differences in serum ferritin concentrations between patients with schizophrenia, bipolar disorder, anxiety, depression compared with controls (40, 41, 51).

These inconsistent results could reflect different responses of ferritin production under different illness states and at different ages since serum ferritin (47, 52) and brain iron and ferritin content increase with aging (53–57), rendering elderly patients with psychiatric disorders vulnerable to oxidative damage (58), ferroptosis (59) and mortality (60) during severe COVID-19.

In this study we aimed to examine whether geriatric COVID-19 patients with comorbid psychiatric conditions demonstrate increased ferritin production as illness severity increases necessitating ICU admission.

## Materials and methods

### Patients and data collection

This study was approved by the Institutional Review Board of the Mayo Clinic and COVID-19 Research Task Force (ID: 21-010940). It included electronic medical records (EMR) review of 927 geriatric patients (≥65 years old) with a confirmed positive SARS-CoV-2 by reverse-transcriptase polymerase chain reaction (RT-PCR) test (nasopharyngeal/oropharyngeal swab specimen), who received medical care at Mayo Clinic Health System from March 1, 2020, through October 1, 2021, and completed a serum ferritin concentration test. For patients completing more than one serum ferritin laboratory test, we selected the first ferritin level at time of admission for consistency and also based on recent reports suggesting the initial ferritin level, rather than change over hospital admission, predicts severity of illness, ICU admission, and need for mechanical ventilation (61). Patient data included demographics, psychiatric and medical comorbidities, and COVID-19 associated hospital course, ICU admission, and mortality.

### Statistical analysis

Continuous variables were summarized using means (SD) and categorical variables using frequencies and percentages. Student’s t-test was used to compare the means of continuous variables and Fisher exact test was used to compare frequencies of categorical variables between the two study groups (with and without psychiatric comorbidities). Factorial ANOVAs were performed to examine the effects of sex, ICU admission, delirium, mortality, and psychiatric comorbidities on serum ferritin concentration covarying for age. Linear regression analysis was utilized to examine the association between serum ferritin and the duration between SARS-CoV-2 positive test and mortality dates in males and females. Analyses were performed with PRISM GraphPad 9 (San Diego, CA) and SPSS V27 software (Armonk, NY: IBM Corp). Results were considered significant at *P* < 0.05.

## Results

### Sample identification

Electronic medical records of Mayo Clinic Health System patients with COVID-19 diagnosis during the interval from March 1, 2020, through October 1, 2021, were examined to identify geriatric patients (≥65 years) with SARS-CoV-2 positive RT-PRC test and serum ferritin values (*n* = 927). We included patients with serum ferritin results up to 14 days before, and up to 30 days after, SARS-CoV-2 positive test (*n* = 628).

### Patient demographics

The study cohort was categorized by the presence (*n* = 212) or absence (*n* = 416) of psychiatric comorbidities. The mean age did not differ between the two groups (79.6 ± 8.6 vs. 79.8 ± 8.4 years), but there were significant sex and race differences. The “no psychiatric comorbidity” group had more males (61.8 vs. 40.1%, *P* < 0.0001), and slightly fewer Caucasians (94.7 vs. 99.1%, *P* = 0.006). Among the no psychiatric comorbidity group (62.5 vs. 48.1, *P* = 0.0006), more reported being married, whereas 33% (vs. 23.1%, *P* = 0.009) of the psychiatric comorbidity group were widowed. Obesity (BMI 30.1–40 kg/m^2^) was reported in 33% of the psychiatric comorbidity group compared to 24% in the no psychiatric comorbidity group (*P* = 0.017) (Table 1).

**TABLE 1:** Demographics.

		No psychiatric comorbidity (*n* = 416)	Psychiatric comorbidity (*n* = 212)	Student’s t-test or Fisher exact test
Age at time of SARS-CoV-2 positive test		79.75 ± 8.6 (66.1–106.0)	79.9 ± 8.4 (65.8–98.4)	*t* = 0.3293, df = 626, *P* = 0.7
Male sex		257 (61.8%)	85 (40.1%)	*P* < 0.0001
Race	Caucasian	394 (94.7%)	210 (99.1%)	*P* = 0.006
	African American	11 (2.6%)	0 (0%)	*P* = 0.019
Non-hispanic ethnicity		403 (96.9%)	205 (96.7%)	*P* > 0.99
Employment status	Retired	349 (83.9%)	187 (88.2%)	*P* = 0.3
	Full time	30 (7.2%)	3 (1.4%)	*P* = 0.001
	Self-employed	18 (4.3%)	9 (4.2%)	*P* > 0.99
	Part time	9 (2.2%)	3 (1.4%)	*P* = 0.7
	Disabled or unemployed	9 (2.2%)	10 (4.7%)	*P* = 0.1
	Missing	1 (0.2%)	0 (0%)	*P* > 0.99
Educational level	≤12 grade or GED	79 (19%)	55 (25.9%)	*P* = 0.0504
	Associate degree	40 (9.6%)	24 (11.3%)	*P* = 0.4
	Some college	25 (6%)	7 (3.3%)	*P* = 0.18
	Bachelor’s degree	47 (11.3%)	16 (7.5%)	*P* = 0.16
	Masters or PhD	28 (6.7%)	9 (4.2%)	*P* = 0.2
	Missing	197 (47.4%)	101 (47.6%)	*P* > 0.99
Marital status	Married	260 (62.5%)	102 (48.1%)	*P* = 0.0006
	Widow	96 (23.1%)	70 (33%)	*P* = 0.009
	Divorced	33 (7.9%)	23 (10.8%)	*P* = 0.2
	Single	23 (5.5%)	14 (6.6%)	*P* = 0.5
	Separated	1 (0.2%)	0 (0%)	*P* = 0.5
	Life-partner	2 (0.5%)	3 (1.4%)	*P* = 0.3
	Missing	1 (0.2%)	0 (0%)	*P* > 0.99
BMI (kg/m^2^)		28.6 ± 7.1 (14.8–63.1)	29.1 ± 6.8 (14.7–48.9)	*t* = 0.9298, df = 624, *P* = 0.3
BMI groups	<18 (kg/m^2^)	8 (1.9%)	6 (2.8%)	*P* = 0.5
	18.1–30 (kg/m^2^)	278 (66.8%)	118 (55.7%)	*P* = 0.006
	30.1–40 (kg/m^2^)	100 (24%)	70 (33%)	*P* = 0.017
	>40 (kg/m^2^)	28 (6.7%)	18 (8.5%)	*P* = 0.4
	Missing	2 (0.5%)	0 (0%)	*P* = 0.5
History of nicotine use		21 (5%)	14 (6.6%)	*P* = 0.4

### Psychiatric and medical comorbidities

Patients in the psychiatric comorbidity group had clinical diagnoses of depression (70.3%), anxiety (42.5%), substance use (9.4%), or schizophrenia/schizoaffective disorders (2.8%). Hypothyroidism and other thyroid disorders were significantly more prevalent in the psychiatric comorbidity group (29.2 vs. 17.8%, *P* = 0.001). Similarly, the percentage of patients with chronic pain and dementia were significantly higher in the psychiatric comorbidity group (20.8 and 11.8% vs. 7.2 and 6.5%, respectively). No other significant differences were found in medical comorbidities between the two groups (Table 2).

**TABLE 2:** Psychiatric and medical comorbidities.

		No psychiatric comorbidity (*n* = 416)	Psychiatric comorbidity (*n* = 212)	Student’s t-test or Fisher exact test
Comorbid psychiatric conditions	Anxiety	0 (0%)	90 (42.5%)	N/A
	Depression	0 (0%)	149 (70.3%)	N/A
	Drug use	0 (0%)	20 (9.4%)	N/A
	Schizophrenia or schizoaffective or bipolar disorder	0 (0%)	6 (2.8%)	N/A
Comorbid medical conditions	**Thyroid**	**74 (17.8%)**	**62 (29.2%)**	***P* = 0.001**
	**Chronic pain**	**30 (7.2%)**	**44 (20.8%)**	***P* < 0.0001**
	**Dementia**	**27 (6.5%)**	**25 (11.8%)**	***P* = 0.03**
	Mild cognitive impairment	13 (3.1%)	14 (6.6%)	*P* = 0.059
	Hypertension	229 (55%)	125 (59%)	*P* = 0.3
	Cardiovascular	200 (48.1%)	88 (41.5%)	*P* = 0.12
	Renal	165 (39.7%)	99 (46.7%)	*P* = 0.10
	Pulmonary	147 (35.3%)	86 (40.6%)	*P* = 0.2
	GIT	138 (33.2%)	79 (37.3%)	*P* = 0.3
	Diabetes	120 (28.8%)	59 (27.8%)	*P* = 0.8
	Neurological	113 (27.2%)	64 (30.2%)	*P* = 0.4
	Atrial fibrillation	109 (26.2%)	50 (23.6%)	*P* = 0.4
	Cancer	88 (21.2%)	40 (18.9%)	*P* = 0.5
	Hepatological	38 (9.1%)	21 (9.9%)	*P* = 0.7
	Organ transplant	14 (3.4%)	7 (3.3%)	*P* > 0.99

Bolded values are the significant values.

### Serum ferritin concentration, hospitalization, ICU admission, delirium, and mortality

Normal serum ferritin in healthy geriatric individuals from Copenhagen (age range 60–93) ranged between 16 and 240 μg/L (62). COVID-19 patients with psychiatric comorbidities had significantly lower ferritin concentration compared to patients without psychiatric comorbidities (570.4 ± 900.1 vs. 744.1 ± 965, *P* = 0.029). However, since the psychiatric comorbidity group had significantly more female patients and there is known sex difference in serum ferritin concentration (63), we compared male and female patients separately within four different ferritin quartiles (<100 μg/L, 100–500 μg/L, 501–1,000 μg/L, and >1,000 μg/L) and found no significant differences (Table 3).

**TABLE 3:** Ferritin concentration, hemoglobin, red and white cell counts, serum iron, interleukin 6 (IL-6) concentrations and hospitalization, ICU admission, delirium, and mortality.

		No psychiatric comorbidity (*n* = 416)	Psychiatric comorbidity (*n* = 212)	Student’s t-test or Fisher exact test
**Ferritin concentration (mg/dL)**		**744.1 ± 965.0 (7**–**13,200)**	**570.4 ± 900.1 (8**–**10,684)**	***t* = 2.177, df = 625, *P* = 0.029**
<100 (mg/dL)	Males	14 (5.4%)	7 (8.2%)	*P* = 0.4
	Females	18 (11.3%)	18 (14.2%)	*P* = 0.4
100–500 (mg/dL)	Males	93 (36.2%)	36 (42.4%)	*P* = 0.3
	Females	87 (54.7%)	74 (58.3%)	*P* = 0.5
501–1,000 (mg/dL)	Males	81 (31.5%)	26 (30.6%)	*P* > 0.99
	Females	33 (20.8%)	26 (20.5%)	*P* > 0.99
>1,000 (mg/dL)	Males	69 (26.8%)	16 (18.8%)	*P* = 0.15
	Females	20 (12.6%)	9 (7.1%)	*P* = 0.16
Hemoglobin (gm/dL)		12.05 ± 2.03 (*n* = 398)	11.83 ± 1.86 (*n* = 197)	*t* = 1.235, df = 593, *P* = 0.2
Red blood cell count (million cell/m^3^)		4.23 ± 0.73 (*n* = 397)	4.18 ± 0.63 (*n* = 196)	*t* = 0.9069, df = 591, *P* = 0.3
White blood cell count (cell/microL)		8.21 ± 9.19 (*n* = 411)	7.52 ± 4.24 (*n* = 208)	*t* = 1.029, df = 617, *P* = 0.3
Serum iron (micromol/L)*		25.55 ± 15.92 (*n* = 58)	32.14 ± 22.47 (*n* = 20)	*t* = 1.549, df = 75, *P* = 0.12
Serum IL-6 (pg/mL)*		58.0 ± 114.0 (*n* = 82)	43.8 ± 60.4 (*n* = 34)	*t* = 0.6856, df = 114, *P* = 0.4
Required hospitalization		395 (95%)	206 (97.2%)	*P* = 0.2
Required ICU admission		73 (17.5%)	36 (17%)	*P* = 0.9
Developed delirium		**60 (14.4%)**	**48 (22.6%)**	***P* = 0.013**
**Mortality**		**109 (26.2%)**	**76 (35.8%)**	***P* = 0.015**

*The results of serum iron and IL-6 are confounded by the small number of patients who had these laboratory values.

Bolded values are the significant values.

Almost all patients (97.2% with psychiatric comorbidity and 95% without) required hospitalization, and approximately 17% in each group required ICU admission. Patients with psychiatric comorbidities had a significantly higher rate of delirium (22.6 vs. 14.4%, *P* = 0.013) and a higher mortality rate (35.3 vs. 27.6%, *P* = 0.015). The interval between SARS-CoV-2 positive test and death was significantly longer in the psychiatric comorbidity group 64.9 ± 93.3 vs. 39.3 ± 54.2 days, *P* = 0.22 (Table 3).

### The effects of sex, ICU admission, and psychiatric comorbidities on serum ferritin

Factorial ANOVA using age as a covariate showed significant effects for sex [*F*(1, 601) = 9.840, *P* = 0.002], ICU admission [*F*(1, 601) = 7.344. *P* = 0.007], and the presence of comorbid psychiatric conditions [*F*(1, 601) = 5.205, *P* = 0.02] on serum ferritin concentration. In addition, we found significant interactions between ICU admission and the presence of comorbid psychiatric conditions [*F*(1, 601) = 4.204, *P* = 0.041].

Pairwise comparisons reveal significantly higher serum ferritin in patients who were male [compared to females: 920.6 ± 76.9 vs. 588.1 ± 72.8], admitted to the ICU [compared to no ICU admission: 897.6 ± 95.8 vs. 611.0 ± 44.7], and without comorbid psychiatric conditions [compared to patients with comorbid psychiatric conditions: 874.9 ± 61.2 vs. 633.7 ± 86.1]. Marked differences between male and female patients in serum ferritin were observed only in those without comorbid psychiatric conditions [male vs. female: 1,098.3 ± 78.4 vs. 651.5 ± 94.4, *P* < 0.001]. No sex difference was observed in serum ferritin among patients with comorbid psychiatric conditions [male vs. female: 742.5 ± 132.0 vs. 524.6 ± 110.8, *P* = 0.2].

**FIGURE 1:**
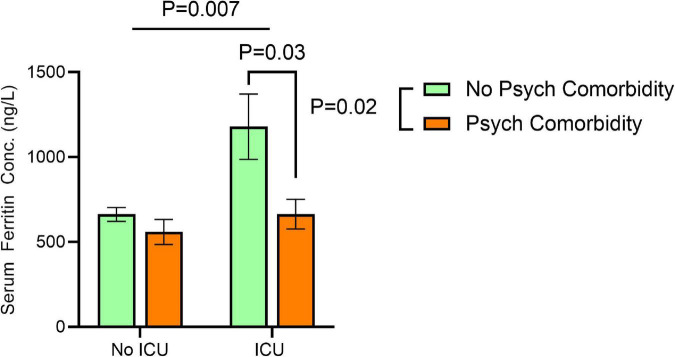
Effects of ICU admission and comorbid psychiatric conditions on serum ferritin concentrations. Significant effects for ICU admission [*F*(1, 601) = 7.344. *P* = 0.007], and the presence of comorbid psychiatric conditions [*F*(1, 601) = 5.205, *P* = 0.02] on serum ferritin concentration correcting for age at time of SARS-CoV-2 positive test by two-way ANOVA. Error bars represent 95% confidence interval.

In addition, robust increase in serum ferritin in ICU admitted patients was only observed in those without comorbid psychiatric conditions [ICU admitted patients without comorbid psychiatric conditions vs. with comorbid psychiatric conditions: 1,126.6 ± 110.7 vs. 668.6 ± 156.5, *P* < 0.001]. Among patients who did not require ICU admission, we did not find any significant difference in serum ferritin between those with and without comorbid psychiatric conditions [598.8 ± 72.1 vs. 623.2 ± 52.8, *P* = 0.6] (Figure 1).

### The effects of mortality and delirium on serum ferritin

**FIGURE 2:**
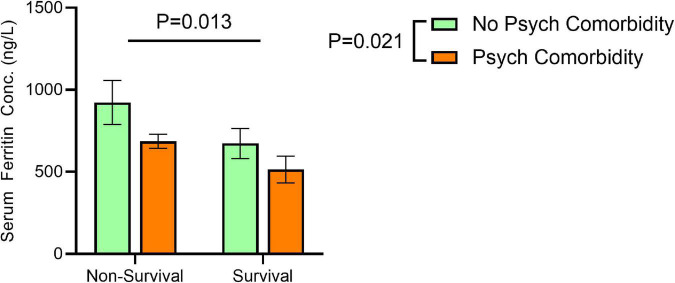
Effects of non-survival and comorbid psychiatric conditions on serum ferritin concentrations. Significant effects for non-survival [*F*(1,620) = 6.080, *P* = 0.013] and psychiatric comorbidity [*F*(1, 620) = 5.33, *P* = 0.02] on serum ferritin, but no interaction between the two factors [*F*(1,620) = 0.2047, *P* = 0.6 on serum ferritin concentration correcting for age at time of SARS-CoV-2 positive test by two-way ANOVA. Error bars represent 95% confidence interval.

We have examined whether serum ferritin differs based on survival (vs. mortality) and the presence (vs. absence) of psychiatric comorbidity Mean serum ferritin was markedly elevated in non-survivors (compared to survivors) without psychiatric comorbidity (922.51 ± 1,401.51 vs. 672.68 ± 797.75), but the difference in serum ferritin between non-survivors and survivors among patients with psychiatric comorbidity was much smaller (686.12 ± 748.86 vs. 513.76 ± 950.64). A significant effect of mortality [*F*(1,620) = 6.080, *P* = 0.013] and psychiatric comorbidity [*F*(1, 620) = 5.33, *P* = 0.02] on serum ferritin was evident, but no interaction between the two factors was found [*F*(1,620) = 0.2047, *P* = 0.6 (Figure 2).

Next, we assessed the association between serum ferritin and the interval between SARS-CoV-2 positive test and death using linear regression and found no significant association in patients with [*F*(1,73) = 0.2454, *P* = 0.6] or without psychiatric comorbidity [*F*(1,106) = 3.273, *P* = 0.073]. Further, we did not find significant effect of delirium on serum ferritin concentration [*F*(1,619) = 0.058, *P* = 0.8] among patients with or without psychiatric comorbidity [*F*(1,619) = 0.05, *P* = 0.8].

### Peak serum ferritin

To examine whether peak serum ferritin concentrations differ between the two groups, we collected all available serum ferritin results and identified the highest value for those with more than one ferritin result and identified the day reaching the peak. About 13–14% had the peak on day 3 (without psych comorbidity) or day 4 (with psych comorbidity) (Supplementary Figure 1). Next, we calculated the percentage change in serum ferritin from baseline to peak and compared the two groups. We found no significant effect of psychiatric comorbidity [*F*(1, 201) = 3.681, *P* = 0.056] or time [*F*(12, 201) = 1.356, *P* = 0.19] on “peak” serum ferritin concentration (Supplementary Figure 2). Finally, we performed a two-way ANOVA (ICU admission and presence or absence of psychiatric comorbidity) and found no significant effect of psychiatric comorbidity on “peak” serum ferritin concentration [*F*(1, 238) = 2.377, *P* = 0.12]. However, it is important here to state that we don’t have solid data to be certain of peak serum ferritin results because (1) we don’t have repeated serum ferritin measurements except for 390 (out of 627), (2) among those we have 114 with only two measures and 276 with 3 or more measurements, and (3) among those with 3 or more measures we have 82 patients with peak serum ferritin as the first ferritin value, so, trying to interpret the results for our available “peak” serum ferritin is not going to be valid.

## Discussion

The results of this study show that in the setting of COVID-19 infection, geriatric patients with and without comorbid psychiatric conditions have high serum ferritin concentrations. However, in critical illness requiring ICU admission, COVID-19 geriatric patients without psychiatric comorbidity had a higher, more robust increase in serum ferritin compared with patients with psychiatric comorbidity. It is possible that ferritin synthesis in these patients is blunted or that there is destruction of ferritin (ferritinophagy) and release of catalytic iron which may cause significant increase in oxidative stress and contribute to mortality. High serum ferritin could also be detrimental if it triggers nuclear receptor coactivator 4 (NCOA4)-mediated ferritinophagy, in which ferritin is degraded and iron is released. Excess iron released from ferritinophagy can increase oxidative stress and promote ferroptosis and cellular damage (64). The brain is highly sensitive to oxidative damage because of its high iron content (65). Failure to augment ferritin synthesis to sequester excessive iron during severe COVID-19 or the destruction of ferritin and rapid release of iron may leave the brain vulnerable to oxidative damage and neurotoxicity. Serum ferritin also plays a role in the regulation of immune response during acute inflammatory states, which may explain the attenuated increase in patients with comorbid psychiatric disorders.

### Ferritin molecule, synthesis, and functions within the cell

Apoferritin (empty ferritin shell) is composed of 24 polypeptide chains arranged into 2 subunits: heavy (H) and light (L) subunits, each coded in a different gene (19). H-ferritin has ferroxidase activity that oxidizes ferrous iron into ferric iron (66). L-ferritin is found mostly in liver, spleen and serum and stores ferric iron up to 4,500 iron atoms can be stored inside the ferritin core (67–70). Tissues with high energy demand such as the brain have high iron content and high production of reactive oxygen species. For that reason, these tissues will have high concentration of H-ferritin to detoxify iron quickly and prevent ferroptosis. The brain has the second highest iron concentration after the liver because of its high metabolic activity and high oxidative stress. Brain iron is found mainly in neurons while ferritin is concentrated in oligodendrocytes (71). Mitochondrial ferritin is close to H-ferritin and has antioxidant properties and protects neurons against ferroptosis (72, 73), while nuclear ferritin protects DNA from oxidative damage (74).

### Serum ferritin

Ferritin is secreted by macrophages, hepatocytes, and other cells into the serum to distribute iron to other cells (75–77). Most serum ferritin is L-ferritin (10). Cells uptake L-ferritin through the Scavenger Receptor Class A member 5 (SCARA5) (78), while H-ferritin receptors are transferrin receptor-1 (79). Serum ferritin is cleared by the liver and its concentration correlates with the concentration of intracellular iron under normal conditions (80). Serum ferritin has low iron content in healthy individuals (1,100–1,200 iron atoms/ferritin) and even lower in patients with inflammation (about 800 iron atoms/ferritin) (8). However, the low number of iron atoms in ferritin is still significantly higher than the iron content in transferrin (2 iron atoms/transferrin) which makes ferritin an important source for rapid delivery of iron at time of stress.

### Severe acute respiratory syndrome coronavirus 2 increases oxidative stress, and the cell responds by upregulating ferritin synthesis

Viruses including SARS-CoV-2 generate a pro-oxidative environment in the host cell to optimize and sustain their replication (7). SARS-CoV-2 infection markedly stimulates nuclear factor-κB (NF-kB) protein expression and activity (81, 82). NF-κB is a central mediator of pro-inflammatory gene induction and functions in both innate and adaptive immune cells. It also targets inflammation not only directly by increasing the production of inflammatory cytokines, chemokines, and adhesion molecules, but also by regulating cell proliferation, apoptosis, morphogenesis, and differentiation. This dysregulated inflammatory response increases oxidative stress (83). In addition, SARS-CoV-2 interferes with the synthesis of antioxidant glutathione (GSH) through consumption of cystine into viral proteins during viral replication (7). Cytokines (TNF-α, IL-1α, IL-1β, IL-6) (84, 85) and oxidative stress upregulate the H-ferritin gene [reviewed in refs (19, 20)]. L-ferritin gene is regulated mostly by high iron content. Inflammation induces ferritin synthesis to divert labile iron into stored iron and reduce the availability of iron that can be used for bacterial growth (86). In addition, serum H-ferritin acts as an immuno-suppressor with some *in vitro* evidence that H- ferritin may directly suppress the differentiation of human B lymphocytes maturing into antibody producing cells (22, 87). Further, ferritin administration in mice protects against *E. coli*-induced sepsis (88).

### Aging and psychiatric comorbidities affect ferritin response to inflammation

Aging is associated with progressive increase in neuronal iron deposition (71), serum ferritin (89, 90) and progressive decline in GSH concentration (91–94), increasing vulnerability to oxidative stress during infection (7). Psychiatric conditions also contribute to the increased vulnerability to oxidative stress. However, production of ferritin under stress conditions is variable. One study showed decreased hippocampal expression of H-ferritin in a mouse model of lipo poly saccharide (LPS)-induced depression (95), while another study showed increased expression of hippocampal ferritin (H and L subunits) in a mouse model of chronic unpredictable mild stress-induced depression (96).

Several factors influence ferritin synthesis in patients with psychiatric disorders such as comorbid substance use. Serum ferritin concentration correlated with alcohol intake (90) and heavy drinking was associated with increased serum ferritin concentration (97). Similarly, opioid use alters ferritin synthesis. μ-opioid agonists specifically elevate neuronal levels of H-ferritin (98–100). Comorbid diabetes and hypothyroidism blunt ferritin response to inflammation since the H-ferritin gene is upregulated by thyrotropin (101), T4 (102), T3 (103), insulin, and IGF-1 (19, 20)]. Sex and racial differences may also impact ferritin synthesis. One study found that black male patients responded to inflammation with a more robust rise in serum ferritin compared to white male patients (104).

High ferritin is reported in severely ill geriatric COVID-19 non-survivors who were hospitalized (*n* = 873) (105) or admitted to the ICU (*n* = 174) (106). Similarly, high ferritin was associated with a 1.7-fold (HR 1.709, 95% CI 1.017–2.871, *p* = 0.043) higher risk of ICU admission or in-hospital death in a cohort of 362 elderly patients (107).

### Sex effect on serum ferritin

We found a significant effect of sex on serum ferritin in those without psychiatric comorbidity. Male patients without psychiatric comorbidity had significantly higher serum ferritin compared to female patients. However, we observed no sex differences in patients with psychiatric comorbidity. Hypothyroidism is more prevalent among psychiatric patients (108) specifically among women (109) and ferritin synthesis is regulated by thyroid hormones (101–103), so it is possible that patients with psychiatric comorbidities, specifically female patients are unable to augment the synthesis of ferritin under the stress of severe infection. Further research is needed to test this hypothesis. Women have significantly lower brain ferritin compared to men (55) suggesting sex-specific influence on iron regulation (110) that may not be related to female sex hormones (111). The reasons behind this sex difference have not yet been fully elucidated.

### Patients with psychiatric comorbidity had significantly higher incidence of delirium and higher mortality

In our study, 22.6% of patients with psychiatric comorbidity developed delirium compared to 14.4% of those without psychiatric comorbidity (*P* = 0.014) and 35.8% died compared to 26.2% in the non-psychiatric comorbidity groups (*P* = 0.015). Delirium is common among COVID-19 patients admitted to the ICU, affecting 61% of patients in one study (112). Delirium is a risk factor for negative impact on recovery from various medical and surgical conditions (113, 114). Furthermore, delirium is associated with high mortality rates during hospitalization (115, 116), high rates of post hospitalization cognitive impairment (117, 118), and mortality (119). Whether disruption in brain iron homeostasis contributes to the development of delirium and overall mortality remains to be further investigated.

Mean serum ferritin was markedly elevated in non-survivors (compared to survivors) without psychiatric comorbidity (922.51 ± 1,401.51 vs. 672.68 ± 797.75), but the difference in serum ferritin between non-survivors and survivors among patients with psychiatric comorbidity was much smaller (686.12 ± 748.86 vs. 513.76 ± 950.64). Rich literature document high serum ferritin among COVID-19 non-survivors (1, 25, 33–39). Our results show that the marked elevation in serum ferritin typically observed in COVID-19 non-survivors, is not present in patients with psychiatric comorbidity who eventually died from severe illness. This new insight into the confounding effect of comorbid psychiatric conditions should be considered when interpreting serum ferritin results in COVID-19 mortality. Further studies to investigate the potential role of ferritin in protecting neuronal integrity during inflammatory-induced stress conditions are needed.

### The confounding effect of metabolic syndrome

The prevalence of diabetes in both groups was similar (about 28%). However, geriatric patients with psychiatric comorbidities had significantly higher rates of obesity (BMI 30.1–40 kg/m^2^) (33 vs. 24%, *P* = 0.017). High ferritin levels are reported in a meta-analysis (*n* = 56,053) to be independently and positively associated with the presence of the metabolic syndrome with an odds ratio higher than 1.73 (120). Unfortunately, we do not have the baseline (before hospitalization) ferritin levels in these patients. Similarly, potential impacts of medication effects were not included in this study.

### Limitations

This study has several limitations including its retrospective design and the documented clinical diagnosis of comorbid psychiatric conditions. Our current results do not answer the question about peak serum ferritin concentration timing or whether it could be different between patients with and without psychiatric comorbidity. Similarly, we are unable to comment on the effect of psychiatric comorbidity, if any, serum iron concentration or inflammatory markers such as IL-6 because of the small number of patients with these laboratory values. In addition, the data collection did not include the extent of substance use, which may have been a confounding factor, particularly in patients developing in-hospital delirium.

## Conclusion

The results of this study show that critically ill, geriatric patients with comorbid psychiatric conditions have attenuated increase in serum ferritin concentration compared to those without comorbid psychiatric conditions. There are at least two possible mechanisms by which to explain this finding. The first could be a failure of psychiatric patients to augment ferritin synthesis due to higher prevalence of hypothyroidism, genetic, or other unknown factors. The second could be that high ferritin-triggered pathways cause rapid release of iron from ferritin, leading to more oxidative damage and cell death. Further research is needed to explore both possibilities and examine the role of psychiatric disorders and ferritin on illness severity and mortality during COVID-19 infection.

## Data availability statement

The original contributions presented in this study are included in the article/Supplementary material, further inquiries can be directed to the corresponding author.

## Ethics statement

The studies involving human participants were reviewed and approved by the Institutional Review Board of the Mayo Clinic and COVID-19 Research Task Force (ID: 21-010940). Written informed consent for participation was not required for this study in accordance with the national legislation and the institutional requirements.

## Author contributions

OA: supervision, drafting of the manuscript, full access to all the data in the study, and takes responsibility for the integrity of the data and the accuracy of the data analysis. OA and AY: concept and design. OA, AY, CE, VN, MA, and YQ: acquisition, analysis, or interpretation of data. All authors: critical revision of the manuscript for important intellectual content, administrative, technical, or material support.

## Supplementary material

Supplementary Figure 1 Time to peak serum ferritin in patients with and without psychiatric comorbidity. About 13 and 14% of patients with and without psychiatric comorbidity respectively reach peak serum ferritin in days 4 and 3 post SARS-CoV-2 positive test.

Supplementary Figure 2 Percentage change from initial to peak serum ferritin concentration in COVID-19 patients with and without psychiatric comorbidity. No significant effect of psychiatric comorbidity on the percentage change in serum ferritin concentration (from initial to peak).
