# Supplementary figures and images for: Attenuated initial serum ferritin concentration in critically ill coronavirus disease 2019 geriatric patients with comorbid psychiatric conditions
Source: Front Psychiatry. 2022 Nov 9;13:1035986. doi: 10.3389/fpsyt.2022.1035986 (PMC9681793; doi:10.3389/fpsyt.2022.1035986)

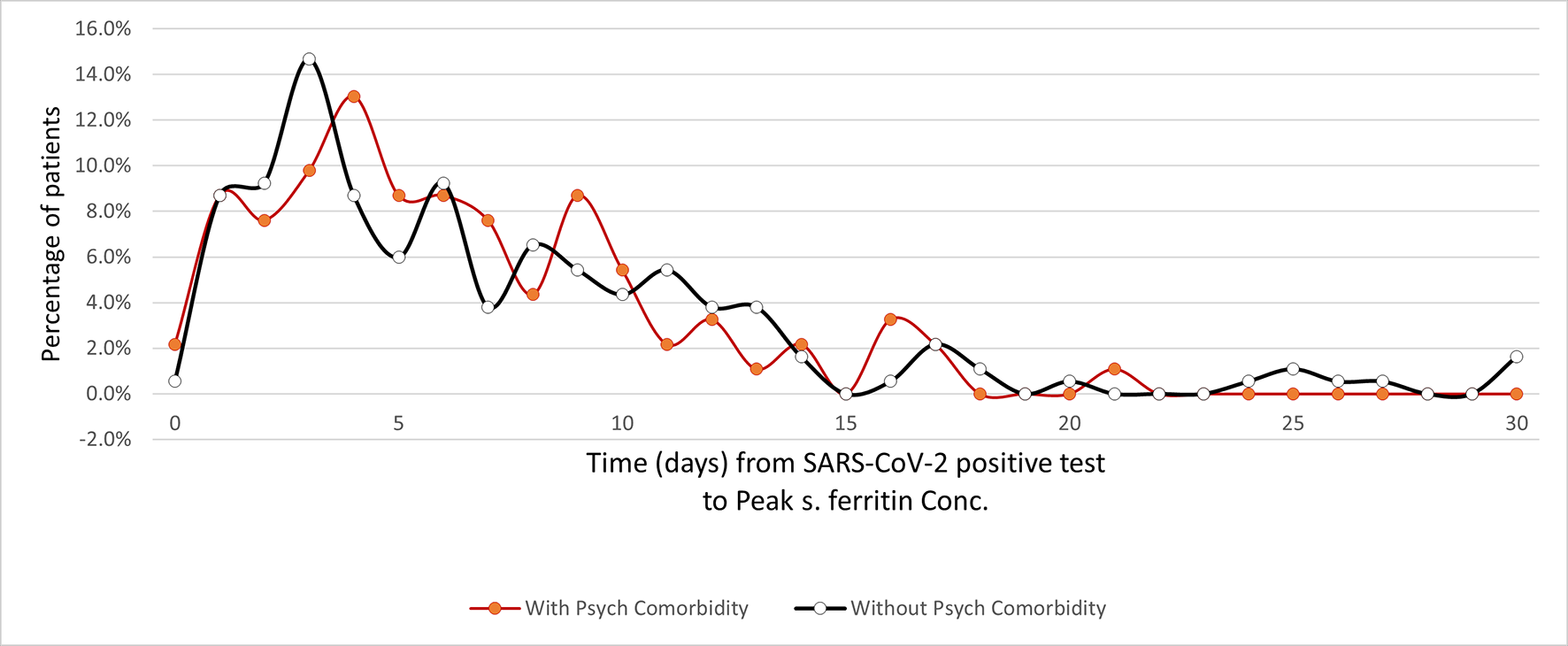

Supplement: Supplementary Figure 1 — Time to peak serum ferritin in patients with and without psychiatric comorbidity. About 13 and 14% of patients with and without psychiatric comorbidity respectively reach peak serum ferritin in days 4 and 3 post SARS-CoV-2 positive test. [file Image_1.tif]

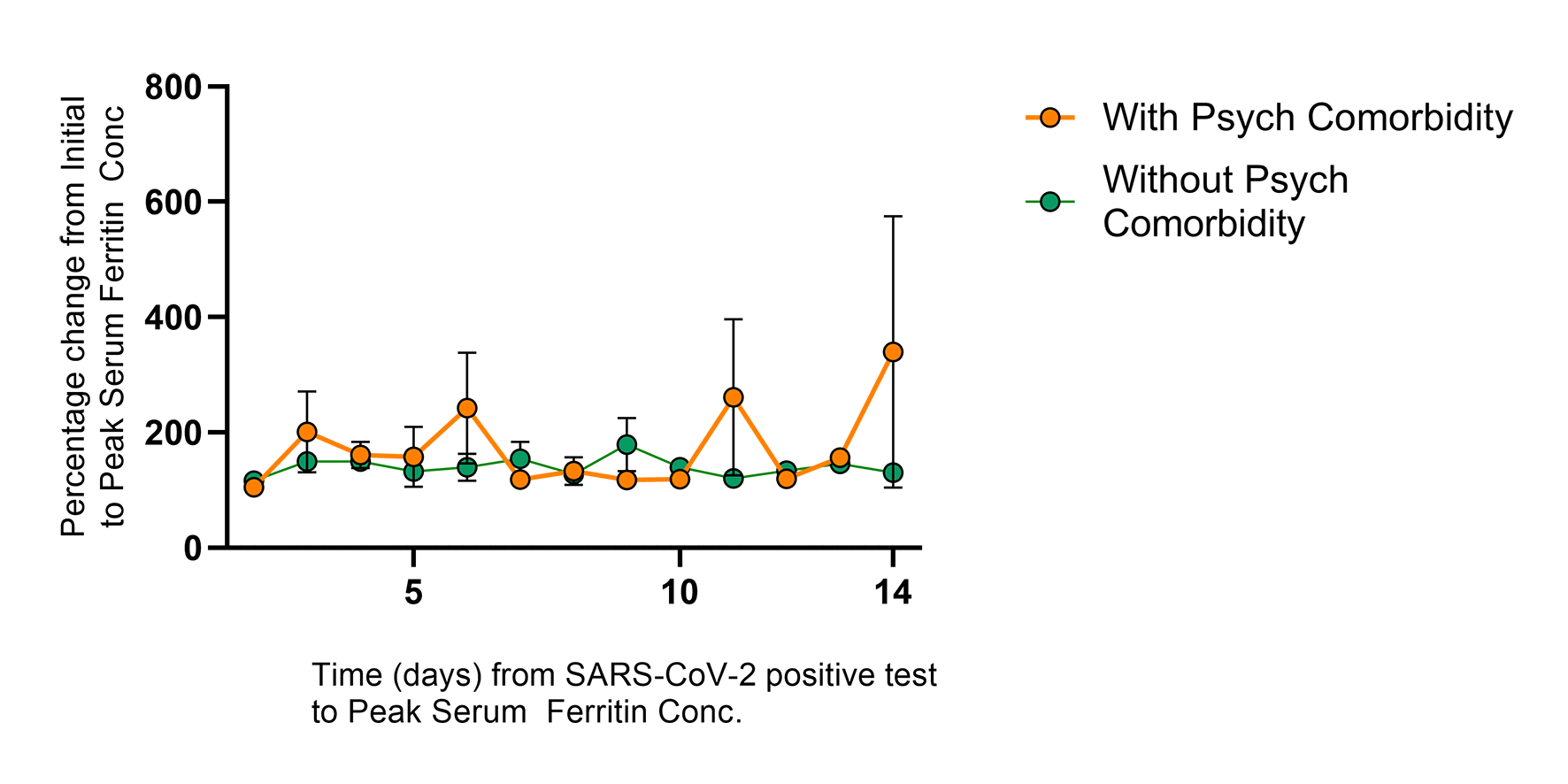

Supplement: Supplementary Figure 2 — Percentage change from initial to peak serum ferritin concentration in COVID-19 patients with and without psychiatric comorbidity. No significant effect of psychiatric comorbidity on the percentage change in serum ferritin concentration (from initial to peak). [file Image_2.tif]
